# Effect of Longitudinal and Transverse Foot Arch on the Position of the Hallux and Fifth Toe in Preschool Children in the Light of Regression Analysis

**DOI:** 10.3390/ijerph19031669

**Published:** 2022-02-01

**Authors:** Ewa Puszczalowska-Lizis, Karolina Krawczyk, Jaroslaw Omorczyk

**Affiliations:** 1 Medical College, Institute of Health Sciences, University of Rzeszow, Warzywna 1a, 35-959 Rzeszow, Poland; 2 District Clinic, Hoffmanowa 8a, 35-026 Rzeszow, Poland; krawczykk04@gmail.com; 3 Institute of Sport, Faculty of Physical Education and Sports, University School of Physical Education, John Paul II Avenue 78, 31-571 Krakow, Poland; JarekOmo@interia.pl

**Keywords:** body posture, foot, health behavior

## Abstract

The aim of the study was to analyze the development of foot structure and the impact of longitudinal and transverse arching of the foot on the position of the hallux and fifth toe in preschool children. The study was carried out among 200 children aged 6 (100 G, 100 B). The research tool was the podoscope CQ-ST. The collected research results were analyzed with the use of the Mann–Whitney U test, Pearson Chi-square test and regression analysis. A statistically significant influence of the heel angle on the value of the varus angle of the fifth toe of the right (girls: *p* = 0.032; boys: *p* = 0.001) and left foot (girls: *p* = 0.004; boys: *p* = 0.005) was found. Boys’ feet are longer and wider than girls’ feet; moreover, they have a lower longitudinal arch. The frequency of longitudinal and transverse arch deformities of the feet, as well as valgus of the hallux and varus of the fifth toe do not depend on gender. In both sexes, decreasing the transverse arch of the foot has an influence on the severity of varus of the fifth toe. Changes in the height of the longitudinal foot arch does not affect the position of the hallux and fifth toe.

## 1. Introduction

The human foot is the base of support and acts as a propelling mechanism, imparting propulsion during locomotion. It acts as a lever in the pre-swing phase and absorbs the rotations of individual segments of the lower limb in the stance phase [1,2,3]. The skeletal system, which is a passive element in the construction of the foot, has a specific architecture, ensuring the maintenance of the body weight and the ability to adapt to changes in the ground, load and movements. This architecture is created, i.e., by a system of longitudinal and transverse arches, which can be compared to springs stretching under load and returning to the initial state due to their specific properties [4,5,6,7]. Toes are important for the proper functioning of the foot. Their proper position enables efficient operation of the remaining segments of the foot. In static conditions, the toes adhere to the ground, and while walking, their strong adhesion relieves the heads of the respective metatarsal bones [8]. Nix et al. [9] emphasized the importance of the metatarsophalangeal joint of the hallux, which serves as a force transfer point in the terminal stance phase. Parcou [10] noted that the anterior metatarsophalangeal hypothenar corresponded on one side to the front of the “posterior static triangle of the foot” and on the other side to the back of the “front dynamic triangle of the foot”. This place concentrates all the impulses that drive the gait. The adaptation to this function is certainly the characteristic structure of the first metatarsal bone, which is the shortest and is much thicker than the others. Di Giovanni and Greisberg [11] emphasized the importance of the heads of the metatarsal bones in carrying the body weight. According to the authors, in a standing position, the head of the first metatarsal is involved in the transfer of about 40% of the body weight, and the rest is distributed over the remaining metatarsal bones. In order to obtain the proper range of motion, straight alignment of the toes as well as proper flexibility and tension of the joint capsules, ligaments and muscles are required. During each step, the hallux is in dorsiflexion, and in the case of its improper positioning or insufficient elasticity of the soft tissues, overloads and disorders of the midphalangeal joint occur. Brügger [12] noted that individual segments of the foot constitute a certain functional unity, therefore, changes in one of them may cause the activation of individually variable compensation mechanisms, leading to changes in the shape of the remaining segments of the foot and disturbances in its functions and, consequently, complications in other parts of the locomotor organ. According to López López et al. [13], López-López et al. [14] and López-López et al. [15], foot pathologies have a negative impact on quality of life.

The preschool period, between 3 and 6 years of age, characterized by a high intensity of ontogenetic developmental changes, is considered the most important from the point of view of foot formation. It is also a critical period with an increased risk of foot defects. The process of arch shaping begins at the age of 3, and the longitudinal and transverse arches are clearly visible at the age of 6, while the Clarke’s angle stabilizes between the ages of 11 and 13, corresponding to the feet of adults [16,17,18,19]. Therefore, the preschool period is of decisive importance for the later health and efficiency of the feet, and getting to know the issues related to the structure of the feet and the interrelationships between the structures that build them is the starting point for proper prophylaxis, examination or correction of deformities.

Understanding the issues concerning the construction of the foot is of key importance, it is the starting point for proper prevention, testing or correction of deformities. The aim of the study was to analyze the development of foot structure and the impact of longitudinal and transverse arching of the foot on the position of the hallux and fifth toe in 6-year-old girls and boys.

Research questions:

1. Do the 6-year-old children demonstrate between genders differences in selected features of foot morphology?
2. Do foot structure features differentiate right and left feet of 6-year-old boys and girls?
3. Does the incidence of longitudinal and transverse arch deformities as well as hallux valgus and the varus deformity of the fifth toe depend on sex?
4. Do the longitudinal and transverse arches affect the position of the hallux and the fifth toe in 6-year-old girls and boys?

## 2. Materials and Methods

The cross-sectional study was carried out among 200 children aged 6 (100 girls and 100 boys) attending randomly selected preschools located at the Rzeszow administrative district. In accordance with the assumption, the 6-year-olds included children aged 6.00 to 6.99 years. The middle of the class totaled 6.5 years. Calendar age, expressed in decimal terms, was the difference between the study date and the date of birth [20].

The inclusion criteria were the calendar age in the range 6.00–6.99 years, being right-handed and right-footed, understanding the instructions that were necessary for the measurement procedures and written informed consent of parents or guardians to participate in the study. The dominant side was established on the basis of comparing the efficiency, speed and precision of performing selected activities with the right and left upper limbs, e.g., holding a spoon while eating, drawing, reaching for an item lying high on a shelf, putting rings into a container, throwing a ball into a box, as well as the lower limb, e.g., kicking a ball, standing on one foot with closed eyes, performing single-leg jumps [21].

The exclusion criteria: lower limb deformities, injury to the musculoskeletal system including lower limbs during the previous 12 months, congenital abnormalities, neurological diseases, prior foot surgery, the use of foot orthoses and also the refusal or unwillingness of the child to cooperate during the implementation of research procedures.

**Figure 1:**
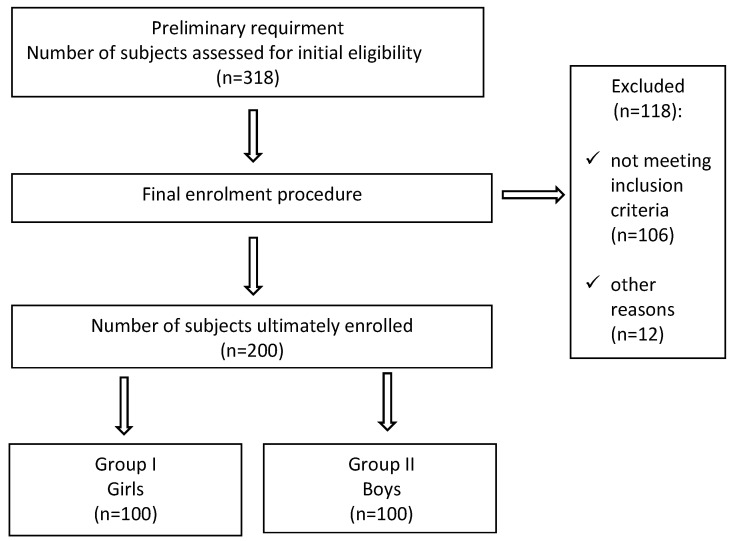
Flow of participants through the study.

The sample size representative for the site was estimated in due consideration of 95% confidence level and a 5% level of admissible error of fraction estimation. Calculations indicated that the sample size should include 318 subjects. After the allocation procedure, it was found that 118 children were excluded from the study protocol due to their non-compliance with the inclusion criteria. The remaining children were divided into 2 equal-sized groups each including 100 individuals on the grounds of the gender. The stages of subject inclusion in the study are presented in Figure 1.

The basic somatic characteristics of the study subjects are presented in Table 1.

The research tool was the podoscope CQ-ST (Electronic System, Ltd., EU). The study included the measurement of the plantar feet surfaces in standing, with even distribution of body weight on both feet. The width and foot angle were natural, unforced. The calculations included the following indices: foot length, foot width, Clarke’s angle (longitudinal foot arch), heel angle (transverse foot arch), hallux valgus angle, the angle of the varus deformity of the fifth toe. The norms according to Lizis [16] were adopted in the interpretation of the above-mentioned indices.

 The examinations were carried out in preschool institutions, in rooms intended for exercise and motor games. In order to ensure the integrity of the research process, all tests were carried out in the morning, using the same measuring instruments operated by the authors. During the study, children were wearing their underwear, without shoes and socks. The study was approved and endorsed by the Bioethics Review Committee of the University of Rzeszow (Approval Ref. No. 2/2/2017). The procedures were carried out in accordance with the Declaration of Helsinki. All participants, their parents or legal guardians were advised of the actual purpose and key principles of the study, as well as on their statutory right to opt out of the study protocol at any stage.

 The Statistica StatSoft, Inc. ver. 13.1 was used to process the test results. The normalcy of distribution of the values was verified by means of the Shapiro–Wilk test. In order to evaluate intergender differences in somatic characteristics and foot structure indices we used the Mann–Whitney U test.

Symmetry index (SI) for individual features describing the foot structure was calculated for each subject according to the formula

$$\mathrm{SI}=\frac{\left|X_{\mathrm{Lf}}-{\;X}_{\mathrm{Rf}}\right|}{0.5\;\cdot \left(X_{\mathrm{Lf}}+X_{\mathrm{Rf}}\right)}\;\cdot 100\%$$

where:

R_f_—value for the right foot,

L_f_—value for the left foot.

The value of SI = 0 indicates full symmetry, while SI ≥ 100% indicates its asymmetry [22].

Qualitative data analysis was performed using the Pearson chi-square test. The influence of independent variables (predictive, explained, such as: Clarke’s angle and heel angle) on the dependent variables (criterial, such as: hallux valgus angle and the angle of the varus deformity of the fifth toe) were estimated on the basis of regression analysis. The results were considered statistically significant if the probability level of the test was lower than the predetermined significance level *p* < 0.05.

## 3. Results

The data in Table 2 show statistically significant differences between the genders in the length of the left foot (*p* = 0.035), as well as the width of the right (*p* = 0.009) and left foot (*p* = 0.009).

The data in Table 3 indicate statistically significant intergender differences in the Clarke’s angle values. This angle was lower in boys, both in the case of the right (*p* < 0.001) and left (*p* < 0.001) foot.

In both girls and boys, the mean and median values of the symmetry index ranged from 0 to 100%. High values of standard deviations for the Clarke’s angle, hallux valgus angle and the angle of the varus deformity of the fifth toe indicate a large variation in this index in the case of the above-mentioned features (Table 4).

The data in Table 5 provide information on the frequency of foot deformities in the examined girls and boys. There were no statistically significant relationships between the frequency of particular types of deformities and sex.

Multiple regression models with two variables explaining (Clarke’s angle and heel angle) the variance of the hallux valgus angle were statistically insignificant for both the right (girls: F = 0.97; *p* = 0.549; boys: F = 0.73; *p* = 0.868) and left feet (girls: F = 0.77; *p* = 0.467; boys: F = 1.55; *p* = 0.215).

The influence of predictive variables on the values of the angle of the varus deformity of the fifth toe was statistically significant for both the right (girls: F = 3.67; *p* = 0.030; boys: F = 6.00; *p* = 0.003) and the left foot (girls: F = 4.61; *p* = 0.012; boys: F = 6.11; *p* = 0.003). These variables together explained respectively: 27%; 33%; 29%; 33% variation in the value of the angle of the varus deformity of the fifth toe because the values of the determination coefficients were, respectively: R^2^ = 0.27; R^2^ = 0.33; R^2^ = 0.29; R^2^ = 0.33. Simple regression showed that only the influence of the heel angle on the value of the varus deformity of the fifth toe was statistically significant for both the right (girls: r_p_ = 0.22; *p* = 0.032; boys: r_p_ = 0.33; *p* = 0.001) and the left foot (girls: r_p_ = 0.29; *p* = 0.004; boys: r_p_ = 0.28; *p* = 0.005). The coefficients of the slope of the regression line for the variables: heel angle and angle of the varus deformity of the fifth toe for the right foot were b = 0.72 in girls, b = 0.95 in boys, and for the left foot b = 0.80 in girls, and b = 0.91 in boys. This means that if the heel angle increases by one unit, the angle of the varus deformity of the fifth toe will increase, respectively, by: 0.72°, 0.95°, 0.80° and 0.91° (Table 6).

## 4. Discussion

In our material, sexual dimorphism of the length of the left foot and the width of the right and left foot were found. Boys’ feet are longer and wider compared to girls’ feet. These data are not in line with the results of Bosch et al. [23] obtained in a group of healthy German children, as well as Delgado-Abellán et al. [24], who did not find gender differences in foot length in 6-year-old Spanish children.

We found that the boys’ feet had a lower longitudinal arch. This suggests that the development of the medial longitudinal arch may be slower in them than in girls. In contrast, Delgado-Abellán et al. [24] did not find any gender differences in the height of the longitudinal arch of the foot in 6-year-olds.

The values of the symmetry index for the analyzed foot structure indices did not exceed 100%, therefore, it is difficult to speak of asymmetry. However, high percentages and values regarding the standard deviation of the symmetry index, with respect to the Clarke’s, hallux valgus angle and the angle of the varus deformity of the fifth toe, are noteworthy. It is difficult to compare the obtained results with the results of other authors as this problem is discussed in a fragmentary way. One of a few studies on this issue was conducted by Vrdoljak et al. [25], who, similarly to our studies, did not observe differences in the length of right and left feet in girls and boys aged 2–7 years old living in the Republic of Croatia. On the other hand, Bari [26] found that there was a significant difference between the length and width of the right and left feet in preschool children from Malaysia.

In this study, the incidence of longitudinal and transverse arch deformation, as well as hallux valgus and varus deformity of the fifth toe do not depend on sex. Similarly, Yin et al. [27], on the basis of studies in children aged 6–13 years, found no relationship between the incidence of flat feet and sex. Šutvajová et al. [28], as a result of the study of children aged 4–6 from Slovakia, found that the ratio of longitudinally flat feet in girls and boys was 1:1.3, respectively. In turn, Bafor and Chibuzom [29], when examining children from Nigeria, noted a greater percentage of hallux valgus in boys.

Taking into account the results of our research, it can be concluded that despite the changes in the foot load pattern typical for the early stages of ontogenesis, the position of the hallux is not strongly disturbed because the values of the hallux valgus angle do not differ significantly from the lower limit of the norm. On the other hand, the obtained results lead to a reflection on the causes of the varus position of the fifth toe in the examined children. The values of this angle in both sexes oscillate around its upper limit. It seems that this condition may be temporary, as a result of the functional deficiency of the muscles responsible for the correct positioning of the fifth toe, as well as the physiological, varus position of the foot at the beginning of its loading and the associated increased pressure on its lateral surface. Moreover, Knapik and Mazur [30] observed a clear tendency in preschool children to deepen the varus of the fifth toe, which was deformed earlier than the hallux. The authors assumed that these changes most often arise as a result of incorrect reactions of the foot with shoes.

An interesting issue is the effect of the longitudinal and transverse arch of the foot on the position of the toes. In our material, the longitudinal arch is not of primary importance for the alignment of the hallux, which is the top of the foot’s dynamic triangle. We also did not notice the influence of the longitudinal arch on the position of the fifth toe, which may be due to the fact that the tensioning of the dynamic medial arch of the foot does not have a direct effect on its lateral edge. Our research has shown the effect of the transverse arch on the position of the fifth toe, both in girls and boys. Increasing the value of the heel angle increases the value of the angle of the varus deformity of the fifth toe. The lack of similar reports excludes the possibility of discussing the obtained results with the conclusions of other authors. It seems, however, that the occurrence of the above-mentioned relationships may be dictated by the conflict of a broadened forefoot with footwear. If the forefoot is flattened and widened, it may be easier to fit the foot into the shoe by tipping or pressing the fifth toe. In this way, the foot, widened due to flattening, can fit into the shoe. Improper placement of the foot in the shoe changes proprioception, therefore, it does not cause discomfort at first. Over time, however, it has an impact on the formation or deepening of deformation, and even microdamage to its delicate structures. This problem requires separate scientific research. The issues related to shoes fitting in terms of width were analyzed by Yurt et al. [31] and Delgado-Abellán et al. [24]. The authors indicated that most footwear designs do not take into account the need to choose different widths for the same length. González Elena and Córdoba-Fernández [32] concluded that when designing ergonomic footwear for children, not only the different digital formulae must be taken into account, but also the position and orientation of the forefoot inside the shoe and its interaction with the shoe tip.

Summing up our research and the reports of other authors, it is necessary to emphasize the need for constant monitoring of the condition of the feet and care for their proper development, especially in the case of children in the period of developmental plasticity. Particularly noteworthy is the position of the fifth toe, which, due to its delicate structure, is prone to deviations from the correct positioning and, therefore, similarly to the hallux, requires early diagnosis. Attention should be paid to the shape of the footwear, including its anterior and lateral surfaces. Properly designed footwear should ensure the comfort of the foot both in static and walking conditions. It is equally important to learn and practice proper foot loading while standing and with locomotion.

Results, as yielded by the present study, make a contribution to further research into this subject, indubitably required with a view to investigating the already established trends even more comprehensively. Given the overall gravity and sheer scale of the issue under study, any subsequent reports would appreciably contribute to its highlighting, while at the same time granting it due prominence in research.

## 5. Conclusions

In 6-year-old children there is sexual dimorphism in the length of the left foot, the width of the right and left foot, and the longitudinal arch of the right and left foot. Boys’ feet are longer and wider than girls’ feet, moreover, they have a lower longitudinal arch. In both sexes, the right and left feet are characterized by symmetry. The frequency of longitudinal and transverse arch deformities of the feet, as well as valgus of the hallux and varus of the fifth toe do not depend on gender. In both sexes, decreasing the transverse arch of the foot has an influence on the severity of varus of the fifth toe. Changes in the height of the longitudinal foot arch does not affect the position of the hallux and fifth toe.

## Figures and Tables

**Table 1:** Comparison of somatic characteristics of the examined girls and boys.

Variable	$\bar{x}$ ± SD	Max–Min	Q_25_	Me	Q_75_	Z_1_	*p*
Body weight (kg)							
Girls	22.59 ± 2.44	28.00–16.40	20.65	22.00	24.00	0.92	0.355
Boys	22.93 ± 2.65	29.00–18.00	21.00	22.65	25.00		
Body height (cm)							
Girls	119.37 ± 4.34	128.00–107.00	117.00	119.00	123.00	−1.53	0.123
Boys	120.47 ± 4.93	131.00–112.00	116.50	120.00	124.50		
BMI							
Girls	15.81 ± 1.17	17.29–13.10	14.81	16.00	16.93	1.16	0.242
Boys	15.74 ± 0.93	17.00–14.00	14.88	15.75	16.67		

Abbreviations: $\bar{x}$ —arithmetical mean value; SD—standard deviation; max—maximum value; min—minimum value; Q_25_—lower quartile; Me—median; Q_75_—upper quartile; Z_1_—value of the Mann–Whitney U test statistic; *p*—probability value.

**Table 2:** Comparison of the length and width of the feet of the examined girls and boys.

Variable		$\bar{x}$ ± SD	Max–Min	Q_25_	Me	Q_75_	Statistics
Foot length (cm)							
R_f_	Girls	17.63 ± 0.91	19.50–15.80	17.00	17.60	18.50	Z_1_ = −1.76 *p* = 0.078
	Boys	17.90 ± 1.02	20.00–15.30	17.00	18.00	18.50	
L_f_	Girls	17.63 ± 0.92	19.50–15.80	17.00	17.60	18.50	Z_1_ = −2.10 *p* = 0.035 *
	Boys	17.93 ± 1.01	20.00–15.30	17.05	18.00	18.50	
Foot width (cm)							
R_f_	Girls	6.88 ± 0.47	8.00–6.00	6.50	6.95	7.20	Z_1_ = −2.61 *p* = 0.009 *
	Boys	7.07 ± 0.54	8.50–5.50	6.65	7.00	7.50	
L_f_	Girls	6.88 ± 0.50	8.00–6.00	6.50	7.00	7.30	Z_1_ = −2.61 *p* = 0.009 *
	Boys	7.08 ± 0.52	8.00–5.50	6.85	7.00	7.50	

Notes: * α = 0.05. Abbreviations: R_f_—right foot; L_f_—left foot; $\bar{x}$ —arithmetical mean value; SD—standard deviation; max—maximum value; min—minimum value; Q_25_—lower quartile; Me—median; Q_75_—upper quartile; Z_1_—value of the Mann–Whitney U test statistic; Z_2_—value of the Wilcoxon test statistic; *p*—probability value.

**Table 3:** Comparison of the features determining the longitudinal and transverse arches as well as the position of the hallux and fifth toe in the examined girls and boys.

Variable		$\bar{x}$ ± SD	Max–Min	Q_25_	Me	Q_75_	Statistics
Clarke’s angle (°)							
R_f_	Girls	37.35 ± 13.48	66.00–5.00	29.00	40.00	48.00	Z_1_ = 4.72 *p* < 0.001 *
	Boys	28.36 ± 13.18	67.00–3.00	18.50	27.00	37.00	
L_f_	Girls	35.48 ± 12.57	60.00–8.00	27.00	36.00	44.50	Z_1_ = 4.76 *p* < 0.001 *
	Boys	26.46 ± 13.55	68.00–0.00	16.00	25.00	35.00	
Heel angle (°)							
R_f_	Girls	17.05 ± 2.03	24.00–13.00	16.00	17.00	19.00	Z_1_ = −0.65 *p* = 0.514
	Boys	17.17 ± 2.11	23.00–10.00	16.00	17.00	18.00	
L_f_	Girls	17.21 ± 2.00	25.00–14.00	16.00	17.00	19.00	Z_1_ = −0.93 *p* = 0.349
	Boys	17.38 ± 1.92	21.00–13.00	16.00	17.00	19.00	
Hallux valgus angle (°)							
R_f_	Girls	3.45 ± 4.17	17.00–0.00	0.00	1.00	6.50	Z_1_ = −1.19 *p* = 0.232
	Boys	3.90 ± 4.03	17.00-(−7.00)	0.00	4.00	6.50	
L_f_	Girls	3.64 ± 3.91	13.00–0.00	0.00	2.00	7.00	Z_1_ = −1.84 *p* = 0.064
	Boys	4.80 ± 4.35	18.00–0.00	0.00	4.00	8.00	
The angle of the varus deformity of the fifth toe (°)							
R_f_	Girls	13.17 ± 6.79	25.00–0.00	8.00	14.00	19.00	Z_1_ = −0.11 *p* = 0.912
	Boys	13.31 ± 6.14	28.00–0.00	9.50	13.00	18.00	
L_f_	Girls	13.58 ± 5.52	28.00–0.00	10.00	14.00	17.00	Z_1_ = −1.68 *p* = 0.092
	Boys	14.64 ± 6.33	27.00–0.00	10.50	16.00	20.00	

Notes: * α = 0.05. Abbreviations: R_f_—right foot; L_f_—left foot; $\bar{x}$ —arithmetical mean value; SD—standard deviation; max—maximum value; min—minimum value; Q_25_—lower quartile; Me—median; Q_75_—upper quartile; Z_1_—value of the Mann–Whitney U test statistic; Z_2_—value of the Wilcoxon test statistic; *p*—probability value.

**Table 4:** Descriptive statistics of symmetry ratios for foot structure indices of the examined girls and boys.

SI (%)	$\bar{x}$ ± SD	Max–Min	Q_25_	Me	Q_75_
Girls					
Foot length	0.64 ± 1.37	5.71–0.00	0.00	0.00	0.00
Foot width	1.39 ± 2.61	8.00–0.00	0.00	0.00	1.37
Clarke’s angle	19.95 ± 25.92	145.95–0.00	0.00	14.07	29.48
Heel angle	5.27 ± 6.80	37.50–0.00	0.00	5.13	6.90
Hallux valgus angle	85.42 ± 87.49	200.00–0.00	0.00	51.31	200.00
The angle of the varus deformity of the fifth toe	40.52 ± 47.99	200.00–0.00	8.00	27.33	49.57
Boys					
Foot length	0.64 ± 1.38	8.45–0.00	0.00	0.00	0.00
Foot width	2.06 ± 3.82	22.22–0.00	0.00	0.00	2.76
Clarke’s angle	32.53 ± 34.28	200.00–0.00	8.33	21.75	44.76
Heel angle	6.94 ± 8.76	50.00–0.00	0.00	5.40	11.60
Hallux valgus angle	72.59 ± 81.35	200.00–(−200.00)	1.43	46.15	133.33
The angle of the varus deformity of the fifth toe	37.33 ± 46.85	200.00–0.00	4.77	22.22	46.15

Abbreviations: $\bar{x}$ —arithmetical mean value; SD—standard deviation; max—maximum value; min—minimum value; Q_25—_lower quartile; Me—median; Q_75__—_upper quartile; SI—symmetry index value.

**Table 5:** The frequency of occurrence of foot deformities of the examined girls and boys.

Variable		Girls	Boys	Total	Chi-Square Test
		n (%)	n (%)	n (%)	
The medial longitudinal arch based on the Clarke’s angle Reference values: 29–49° for girls; 20–44° for boys [16]					
R_f_	Normal foot	66 (66.0)	62 (62.0)	128 (64.0)	χ^2^(2) = 0.35 *p* = 0.838
	Flat foot	23 (23.0)	26 (26.0)	49 (24.0)	
	High arched foot	11 (11.0)	12 (12.0)	23 (12.0)	
L_f_	Normal foot	61 (61.0)	60 (60.0)	121 (61.0)	χ^2^(2) = 1.17 *p* = 0.555
	Flat foot	26 (26.0)	31 (31.0)	57 (28.0)	
	High arched foot	13 (13.0)	9 (9.0)	22 (11.0)	
Transverse arch based on the heel angle Reference values: 15–18° [16]					
R_f_	Normal foot	64 (64.0)	68 (68.0)	132 (66.0)	χ^2^(2) = 0.36 *p* = 0.836
	Flat foot	27 (27.0)	24 (24.0)	51 (25.0)	
	High arched foot	9 (9.0)	8 (8.0)	17 (9.0)	
L_f_	Normal foot	66 (66.0)	62 (62.0)	128 (64.0)	χ^2^(2) = 0.40 *p* = 0.818
	Flat foot	27 (27.0)	31 (31.0)	58 (29.0)	
	High arched foot	7 (7.0)	7 (7.0)	14 (7.0)	
Setting of the hallux based on the hallux valgus angle Reference values: 0–9° [16]					
R_f_	Normal setting	90 (90.0)	93 (93.0)	183 (92.0)	χ^2^(1) = 0.58 *p* = 0.447
	Hallux valgus	10 (10.0)	7 (7.0)	17 (8.0)	
L_f_	Normal setting	90 (90.0)	88 (88.0)	178 (89.0)	χ^2^(1) = 0.20 *p* = 0.651
	Hallux valgus	10 (10.0)	12 (12.0)	22 (11.0)	
Setting of the fifth toe based on the angle of the varus deformity of the fifth toe Reference values: 0–9° [16]					
R_f_	Normal setting	33 (33.0)	25 (25.0)	58 (29.0)	χ^2^(1) = 1.55 *p* = 0.212
	The fifth toe varus deformity	67 (67.0)	75 (75.0)	142 (71.0)	
L_f_	Normal setting	22 (22.0)	19 (19.0)	41 (21.0)	χ^2^(1) = 0.27 *p* = 0.599
	The fifth toe varus deformity	78 (78.0)	81 (81.0)	159 (79.0)	

Abbreviations: R_f_—right foot; L_f_—left foot; n—number of subjects; %—percent of subjects; χ^2^—value of the chi-square test statistic; *p*—probability value.

**Table 6:** Regression models in which the independent variables are the indices of longitudinal and transverse arching of the foot.

Variable		R	R^2^	F	*p*	b	r_p_	*p*
Hallux valgus angle (°) of the right foot								
Girls	Clarke’s angle (°)	0.21	0.49	0.97	0.549	−0.03	−0.09	0.353
	Heel angle (°)					0.39	0.19	0.058
Boys	Clarke’s angle (°)	0.12	0.46	0.73	0.868	0.03	0.11	0.278
	Heel angle (°)					0.10	0.05	0.589
Hallux valgus angle (°) of the left foot								
Girls	Clarke’s angle (°)	0.12	0.02	0.77	0.467	0.01	0.03	0.804
	Heel angle (°)					0.24	0.12	0.225
Boys	Clarke’s angle (°)	0.18	0.03	1.55	0.215	−0.05	−0.16	0.118
	Heel angle (°)					−0.16	−0.07	0.489
The angle of the varus deformity of the fifth toe (°) of the right foot								
Girls	Clarke’s angle (°)	0.27	0.07	3.67	0.030 *	0.08	0.16	0.121
	Heel angle (°)					0.72	0.22	0.032 *
Boys	Clarke’s angle (°)	0.33	0.11	6.00	0.003 *	−0.02	−0.04	0.645
	Heel angle (°)					0.95	0.33	0.001*
The angle of the varus deformity of the fifth toe (°) of the left foot								
Girls	Clarke’s angle (°)	0.29	0.09	4.61	0.012 *	−0.02	−0.05	0.622
	Heel angle (°)					0.80	0.29	0.004 *
Boys	Clarke’s angle (°)	0.33	0.11	6.11	0.003 *	0.08	0.18	0.072
	Heel angle (°)					0.91	0.28	0.005 *

Notes: * α = 0.05. Abbreviations: R—coefficient of multiple correlation; R^2^—coefficient of determination; F—value of the Fisher–Snedecor test statistic; b—coefficient of slope of the regression line; r_p_—partial correlation; *p*—probability value.

## Data Availability

Even though the source datasets analyzed in this article are not publicly available, they may be made available to the researchers by the corresponding author upon reasonable request, subject to the applicable legal restrictions in place.
